# Testing Homes for Potential Sources of Lead Exposure as a High‐School Science Project

**DOI:** 10.1029/2021GH000498

**Published:** 2021-11-01

**Authors:** Evan M. Sefchick, Daniel Dusevic, Jack R. Dougherty, Andrew Terraciano, Tyler Ellis, Alexander van Geen

**Affiliations:** ^1^ Pelham Memorial High School Pelham NY USA; ^2^ Lamont‐Doherty Earth Observatory of Columbia University Palisades NY USA

**Keywords:** lead exposure, soil, paint, water, citizen‐science

## Abstract

High‐school students tested soil, paint, and water for lead (Pb) in a total of 80 houses in their town of Pelham, New York, where blood‐Pb data indicate relatively high levels of child exposure. All the samples were tested in the laboratory using established procedures but this was preceded by testing of soil and paint in the field with a kit by the students. The total Pb concentration in 32 of the 159 soil samples that were collected exceeded 400 ppm, the EPA standard for bare soil in residential areas where children play. Only 4 of the 118 tap water samples that were collected contained over 15 ppb Pb, with the data showing that flushing for 2 min clearly lowered Pb concentration further across the board. The highest risk of child exposure may be posed by old Pb‐based paint, however, which was detected in 9 of the 48 samples that were tested. Residents were also the least willing to let the students test or sample their paint. High‐school students could help reduce exposure in the many towns where child blood‐Pb levels remain high today by identifying sources and, while doing so, learn about environmental science and measurement from this hands‐on experience.

## Key Points

- By relying on local links, high‐school students gained access to and tested 80 houses in their neighborhood for lead in soil, paint, and water
- Soil, paint, and water samples exceeded regulatory standards for lead in 19%, 19%, and 3% of samples, respectively
- A simulation based on test results indicates that a single lead exceedance is more likely to come with another exceedance in the same house

## 1 Background

Child exposure to lead (Pb), a well‐established neurotoxin, has declined dramatically in many parts of the world in recent decades, although not necessarily in developing countries (Tong et al., 2000). Relative to the current reference level set by the Centers for Disease Control (CDC) and Prevention, for instance, the proportion of 1–5 year olds with blood‐Pb concentrations exceeding 5 μg/dL in the United States declined from >99% in 1976–1980 to 33% in 1988–1992, 10% in 1999–2000, and finally 0.6% in 2013–2014 (Pirkle et al., 1994; Tsoi et al., 2016). This decline has been attributed to the phasing out of tetraethyl‐Pb additives in gasoline in the two decades leading up to their complete ban in 1996, the banning of Pb‐based paint for residential use in 1978, and an end to the manufacturing of Pb‐soldered food and soft drink cans in 1991 (Pirkle et al., 1994).

In spite of the decline in Pb exposure, at least 1 million children in the United States are still exposed today to Pb levels that may impact their behavior in school, their chances of graduating from high‐school, the risk of juvenile incarceration, and lifetime earnings (Aizer & Currie, 2019; Grosse et al., 2002; Roberts et al., 2017; Tsoi et al., 2016). The main sources of ongoing Pb exposure are known but the specifics vary considerably across regions, towns, and even from one household to the next (Landrigan et al., 2018). The importance of preventing infants from ingesting Pb‐based paint chips and dust has long been recognized but can still be a serious issue in older houses (Dixon et al., 2009; Lanphear et al., 1998). Elevated Pb levels in tap water in a number of U.S. cities documented in Washington DC, Flint, Michigan, and more recently Newark, New Jersey point to another potentially significant and variable source (Pieper et al., 2017). There is also considerable evidence of a link between child Pb exposure and playing in contaminated soil and/or dust from soil, although this is less frequently recognized by health departments (Filippelli et al., 2018; Laidlaw et al., 2016; Lanphear et al., 1998; Mielke et al., 2017).

**Figure 1:**
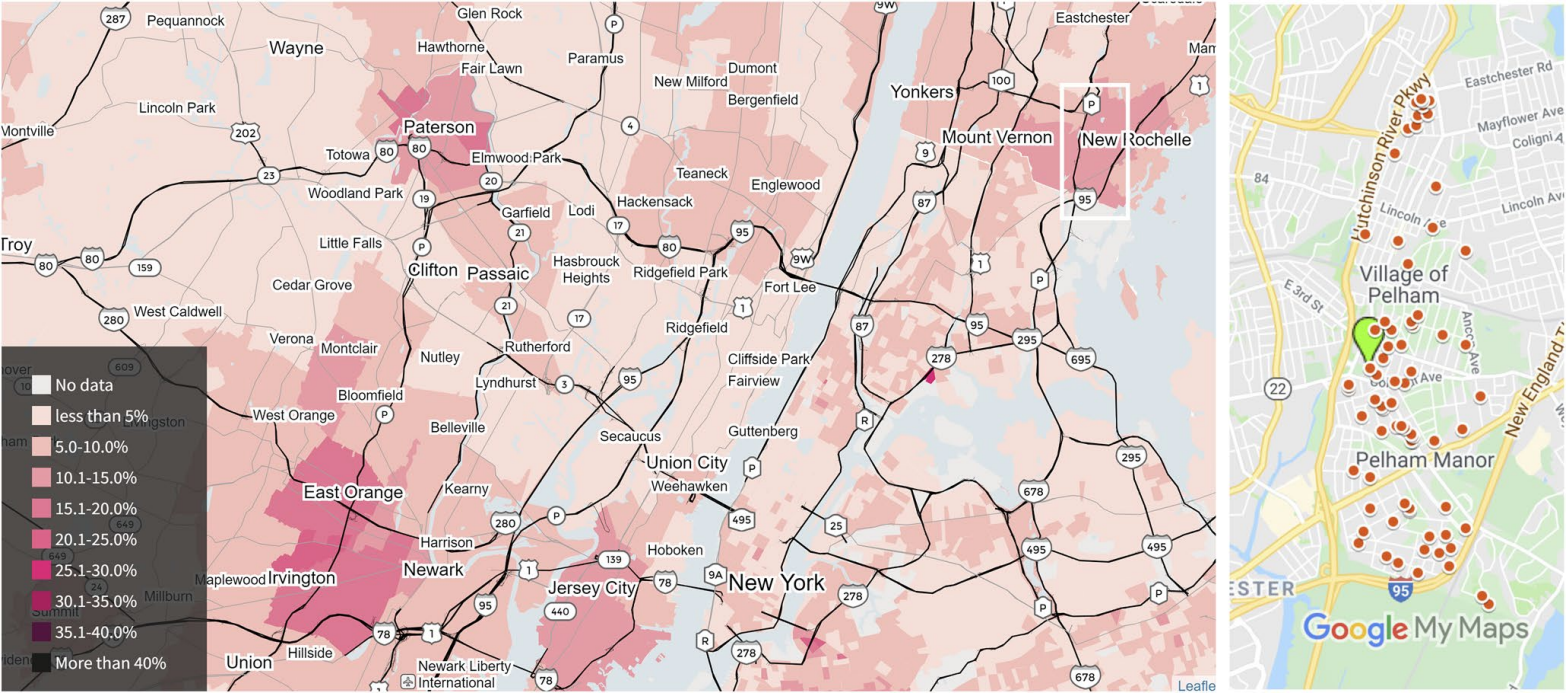
Maps showing (a) The proportion of 1–5‐year olds tested in the metropolitan New York area whose blood‐Pb level exceeded 5 μg/dL in 2005–2015 (from https://www.reuters.com/investigates/graphics/lead-water/en/) and (b) A close‐up of the Pelham area, outlined by a white rectangle in panel (a), showing the locations of the 80 houses from which samples were collected for this study (red circles) and of Pelham Memorial High School (green marker).

Perhaps surprisingly, the most detailed United States‐wide map of child blood‐Pb concentrations has not been issued by the CDC but by a team of investigative reporters at Reuters who had to file Freedom of Information Act requests in each state to gain access to the data (https://www.reuters.com/investigates/graphics/lead-water/en/). The map shows elevated levels in many cities across the northeast and the midwest in particular. A large‐scale survey of the United States shows a similar pattern in soil Pb concentration, with typically higher concentrations toward the surface that integrated decades of deposition from various sources (Smith et al., 2019). The motivation for the present study was two‐fold: (a) the presence of a hot spot of Pb exposure in the Reuters map located around the towns of Mount Vernon and New Rochelle NY, with blood‐Pb levels in 10%–12% of 1–5 year olds exceeding 5 μg/dL between 2005 and 2015 (Figure 1) and (b) the interest of Pelham Memorial High School teachers and students located within this region to investigate the origin of the hot spot. The objectives of the study were therefore to investigate the origin of a local hot‐spot in Pb exposure by following a somewhat unusual approach to sampling and testing soil, paint, and water as part of a high‐school science project that spanned three summers.

## 2 Methods

The sampling of a total of 80 houses was conducted during summer 2018 (27 houses), 2019 (44), and 2020 (9). Initially, the focus was on the students' own houses and that of their friends and neighbors. Over time, additional families were recruited through various school and community groups for better coverage of the town (Figure 1). The field data, including informed consent from a resident from each house, were recorded on smartphones using Kobo Collect (https://www.kobotoolbox.org/). Residents of 38 houses were able to report the decade during which it was built. All but eight houses within this group were built before 1931 (Table 1). A total of 139 soil samples and 48 paint samples were tested by X‐ray fluorescence (XRF) in the laboratory. Most of the soil samples (97) and paint samples (42) were also screened for Pb with a kit shortly after collection (Tables 2 and 3). In addition, pairs of kitchen tap water samples, drawn first thing in the morning after water had been sitting in the pipes overnight, and after flushing for 2 min from 59 houses (Table 5) were analyzed in the laboratory by inductively coupled plasma mass spectrometry (ICPMS). All the results were communicated in writing by mail or email to the residents of the tested houses along with some context and recommendations.

**Table 1:** Overview of Number of Houses and Samples Collected

Year built	No. houses	Soil XRF samples	Paint XRF	Water ICPMS
<1920	9	16	12	14
1921–1930	21	38	17	40
1931–1940	3	2	0	6
1951–1960	4	6	3	4
>1980	1	2	0	2
Unknown	42	75	16	52
Total	80	139	48	118

**Table 2:** Soil XRF (ppm) and Kit Test Results

XRF Pb	XRF no.	XRF and kit	Low	Medium	High
≤400	107	77	72	5	0
400–1,200	26	17	9	3	5
>1,200	6	3	0	0	3

**Table 3:** Paint XRF Results

XRF Pb (ppm)	No. outside	No. inside
≤5,000	17	22
>5,000	4	5

### 2.1 Soil

High‐school students were shown how to collect surface soil samples to ∼2 cm depth with a stainless steel spoon and sieve them (∼1 mm mesh size) on location into 20 mL scintillation vials. The first sample was usually collected near a wall of the house in the front yard and the second in the backyard, targeting areas where children might play. A total of 139 soil samples out of a potential total of 160 that could have been collected from 80 houses were returned for analysis to the Environmental Sciences laboratory at Barnard College, Columbia University (Tables 2 and 3).

The sieved soil samples were screened for hazardous levels of Pb using a field procedure (Landes et al., 2019). The kit is derived from a standard U.S. Environmental Protection Agency (EPA) method to assess the level of bioaccessible Pb in soil, as a proxy for the amount of Pb that might enter the bloodstream of a child ingesting soil. The kit categorizes soil samples as low, medium, or high in terms of their concentration of bioaccessible Pb based on a visual indicator, with the thresholds between the categories corresponding roughly to 400 and 1,200 ppm total Pb. These levels correspond to the current standard for Pb in bare soil in residential areas where children play and where children do not play, respectively.

Without further processing, the fine fraction of the soil was analyzed in the inverted vials through plastic cling wrap using a handheld Innov‐X (now Olympus) Delta Premium X‐ray fluorescence analyzer mounted in a bench‐top stand. The XRF's internal calibration was confirmed by bookending each round of analyses with Standard Reference Material soil 2711a from the U.S. National Institute of Standards and Technology. The average of 1, 480 ± 40 mg/kg (SD, *n* = 4) obtained for Pb was consistent with the certified value of 1,400 ± 10 mg/kg and the data are therefore reported without further adjustment.

### 2.2 Paint

Only 48 paint samples out of a possible total of 160 were returned to the laboratory in 20 mL scintillation vials and were also tested by XRF in the laboratory. Without further treatment or crushing, the chips were analyzed in the inverted vials through plastic cling wrap, with again a NIST 2711a analysis at the beginning and end of each run. The number of samples is small because residents were often reluctant to have their paint tested or sampled, especially if this paint was new and not peeling. The samplers therefore targeted old peeling paint, first outside and then inside the house, in areas where children might play. Most of the paint samples (42) were screened for Pb at the time of collection with the widely distributed 3M LeadCheck kit (Tables 2 and 3).

### 2.3 Tap Water

After soil and paint testing, the residents of each house were left with two acid‐cleaned 250 mL Nalgene bottles to collect a pair kitchen tap water samples on the next morning. The high‐school students picked up both bottles from residents of 59 houses (Table 5). The samples were acidified to 1% Optima HCl in the laboratory, allowed to stand for at least 24 hr, and analyzed for Pb by inductively coupled plasma mass spectrometry (Cheng et al., 2004). The detection limit of the method was 0.1 ppb and long‐term reproducibility on the order of 5% based on the repeated analysis of consistent standards with each batch. Analysis of NIST water standard 1640a resulted in an average of 12.09 ppb, which is consistent with the certified value of 12.005 ppb. Analysis of NIST water standard 1643f resulted in an average of 19.00 ppb, which is close to the certified value of 18.303 ppb.

### 2.4 Statistical Analysis

Many of the houses were missing some samples, particularly in the case of paint. This makes it difficult to determine if one type of contamination is correlated with another. To simulate what the results might have looked like if the distribution of exceedances had been entirely independent of each other, the proportion of exceedances and missing data for each category, that is, soil, paint, and water, were randomly re‐assigned across 80 hypothetical houses a total of 10,000 times in R Studio. The results from this simulation were then compiled in the form of histograms showing the number of houses without any exceedances and one or multiple exceedances.

## 3 Results

### 3.1 Soil

Soil Pb concentrations measured by XRF indicate that 107 (77% of 139) samples met the EPA standard of 400 ppm Pb for bare soil in residential areas where children play (Table 2). Another 26 (19%) soil samples did not meet that standard but contained less than 1,200 ppm Pb. The Pb concentration in the remaining six samples (4%) ranged from 1,300 to 4,500 ppm.

Among the 77 soil samples with ≤400 ppm Pb that were also screened with the field procedure for bioaccessible Pb (Table 2), the kit reading was low for 72 (94%) and medium for another five samples (6%). Within the 17 samples in the intermediate category containing 400–1,200 ppm Pb, kit readings were low for 9 (53%), medium for 3 (18%), and high for 5 (29%). All three samples with >1,200 ppm Pb that were also screened with the kit produced a high reading.

### 3.2 Paint

Based on the federal definition of 5,000 ppm Pb, 9 (19%) out of 48 paint samples that were analyzed by XRF were lead‐based paint (Table 3). The proportion was essentially the same for indoor and outdoor paint. Half of the eight samples of lead‐paint that were tested with the 3M kit resulted in a high reading (Table 4), with the remaining readings categorized as medium (1) and low (3).

**Table 4:** Paint XRF and Kit Results

XRF Pb (ppm)	No. XRF & kit		
	Low	Medium	High
≤5,000	30	3	1
>5,000	3	1	4

### 3.3 Tap Water

A total of 3 (5%) of the 59 first‐draw and 1 (2%) of the 2‐min flush samples of tap water did not meet the EPA standard for Pb in drinking water of 15 ppb (Table 5). The Pb concentration in the water sample not meeting the standard ranged from 16 to 57 ppb. The mean Pb concentrations in the 59 first‐draw samples of 5.2 ppb (SD 8.1) was significantly higher than the mean of 1.5 ppb (SD 2.8) in the 59 2‐min flush samples (*t*‐test for two sample means assuming unequal variance: *t*(71) = 3.35, *p* = 0.001). When the total 4 samples with >15 ppb Pb are excluded, the difference between the mean for the remaining 56 first‐draw samples of 3.8 ppb (SD 3.4) and the 58 2‐min flush samples of 1.2 ppb (SD 2.0) is maintained (*t*(88) = 4.9, *p* < 0.001). This reflects the considerably higher proportion of tap water samples with 5–15 ppb Pb among first‐draw samples (18 out 59) compared to 2‐min flush samples (2 out of 59).

**Table 5:** Tap Water Results

Pb (ppb)	First draw	2 min flush
≤5	38	56
5–15	18	2
>15	3	1

## 4 Discussion

The proportion of exceedances of either of the three federal standards for Pb in soil, paint, and first‐draw water, may seem modest at 19%, 19%, and 5%, respectively. However, the demonstrated long‐term impacts of child exposure from ingesting soil, paint, or water elevated in Pb mean even such moderate proportions are a serious issue. Although the current reference level of blood‐Pb in children is 5 μg/dL, the effects of Pb on intellectual function have been demonstrated at lower levels and no level can therefore be considered safe (Lanphear et al., 2005).

### 4.1 Implications for Blood‐Pb

The soil Pb concentrations encountered during this study are somewhat lower than measured during a survey conducted across New York City reporting over two‐thirds of samples with >400 ppm Pb and concentrations as high as 9,000 ppm (Cheng et al., 2015). The proportion of samples with Pb concentrations would be even higher if a lower standard considered by EPA is adopted (Henry et al., 2015). In the case of soil containing 1,050 ppm Pb, the average for the 32 samples with >400 ppm Pb in the present study, the EPA's Integrated Exposure Uptake Biokinetic (IEUBK) model for Pb in children in contact with the soil predicts an average increment of 5.1 μg/dL to the child's blood Pb level from this source alone (White et al., 1998). The same model predicts that the blood‐Pb concentration of 52% of children would exceed the CDC's current reference level. Yard soil and street dust have also been shown to contribute about two‐thirds of the Pb present in house dust (Adgate et al., 1998). Assuming no other Pb contribution and therefore a house‐dust concentration of 700 ppm based on average soil for the exceedances, the IEUBK model predicts that an average blood‐Pb increment of 4.3 ppb with 37% of blood‐Pb levels >5 ppb from dust alone.

For comparison, the IEUBK model predicts an average increment of only 1.3 μg/dL and less than 0.2% above the blood‐Pb threshold of 5 μg/dL, assuming no other sources than drinking water containing 15 ppb Pb. Fortunately, average Pb levels in tap water were in most cases considerably lower. Exceedance may have been in the particulate form and due to corrosion of Pb pipes or fittings (Triantafyllidou et al., 2007). Whereas the one case where water Pb concentration increased with flushing could be a case of exchanging the two sample bottles, detailed studies have shown that flushing does not always lower water Pb levels at the tap (Katner et al., 2018; Pieper et al., 2015).

The consequences of Pb‐based paint ingestion are hard to gauge because they are highly dependent on the dilution of dust from Pb‐paint with other low‐Pb house dust and yard soil, which is likely to vary considerably room to room. In the extreme case of house dust containing 5,000 ppm Pb, fortunately an unlikely scenario, the IEUBK model predicts an enormous increment of 21 μg/dL in a child's blood Pb level, with blood‐Pb levels of over 99% of children exceeding 5 μg/dL.

The available set includes only one house whose date of construction was recorded and was built after the Pb‐based paint ban of 1978. This house showed no soil or water exceedances (paint was not tested, but extremely unlikely to have been high). The results for the remaining houses show no obvious linkage between exceedances of regulatory standards for soil, paint, and water. Evaluating such connections is complicated, however, by missing data for many of the sampled houses for one or several potential sources of exposure (Table 1).

### 4.2 Correlations Among Exceedances

Overall, only 50 out of 80 sampled houses showed no exceedances of federal standards for any of the samples that were collected. However, a complete set of two samples each of soil, paint, as well as water was obtained and analyzed for only four of the houses without any exceedances. A considerable number of exceedances therefore probably went undetected, especially in the case of paint which was the only missing information for 23 of the remaining 46 houses with a partial set of analyses. The missing data makes it more difficult to establish correlations between different types of exceedances.

**Figure 2:**
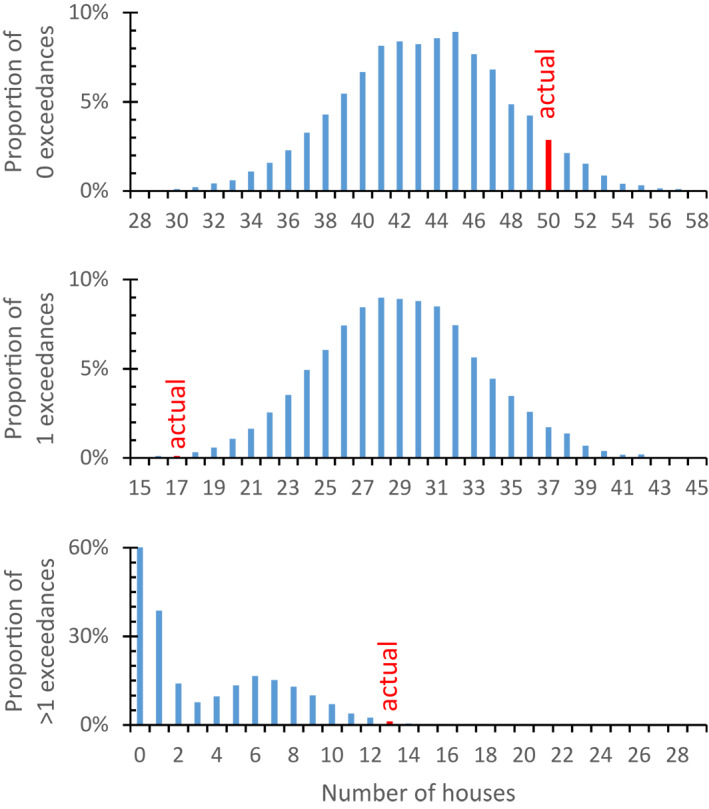
Comparison of the actual occurrences of 0, 1, and 2–3 exceedances within the 80 tested houses with the outcome of 10,000 simulations of the same proportions of exceedances and missing data within each category re‐assigned randomly among hypothetical sets of 80 houses.

To simulate what the results might have looked like if the distribution of exceedances had been entirely independent of each other, the proportion of exceedances and missing data for each category, that is, soil, paint, and water, were randomly re‐assigned across 80 hypothetical houses a total of 10,000 times. The observed total of 50 houses without any exceedance as well as the 13 houses with two or three exceedances, respectively, are at the high end of the distribution resulting from the simulations (Figure 2a). In contrast, the observed 17 houses with a single exceedance are at the very low end of the range resulting from the simulations. This indicates that there are more houses without any or multiple exceedances than if these exceedances had been entirely uncorrelated. The occurrence of a single exceedance in the sample set was much rarer than it would have been if exceedance had been entirely uncorrelated across categories. In other words, the chances of an additional exceedance for a given house are greater if there is one than if there is none.

### 4.3 Importance of Student Participation

Exceedance of Pb standards may not be frequent in Pelham, but combined with even the somewhat out‐of‐date blood‐Pb data, they still warrant the testing of every home, especially in the case of paint. Local government already offers free water testing in Westchester County, but to our knowledge not free testing of paint or soil. Such testing is typically conducted by certified contractors paid by individual homeowners. Some homeowners may not be willing to pay for this service, others may not even want to know for fear that such information would reduce the value of the house without fully realizing the consequences for their own young children.

This is where the participation of students from a local high‐school came into its own. The students started by testing their own houses after discussing the project with their parents and then expanded their survey to the houses of classmates and friends, occasionally advocating for such testing when needed. Local participation generates a level of trust that helped overcome the reluctance of some residents to have their home tested. Field activities during the last summer of the project were limited by the COVID‐19 pandemic but recruitment proceeded through various local groups of parents. Over the years, all homes within the school's catchment area could be reached while providing an opportunity for students to become excited about science and discovery through their own environmental sampling, measurements, and mapping their results. Given the particularly high risk associated with Pb paint, future campaigns should try to emphasize the importance of these measurements whenever residents seem reluctant.

Similar citizen‐science efforts to test Pb in the urban environment are underway elsewhere and could be grown throughout the United States under high‐school programs as well (Filippelli et al., 2018; Tighe, Knaub, et al., 2020). Since households seem to be more motivated by testing their water, paint and soil testing could perhaps be combined with a new procedure for concentrating Pb in water onto a filter and analyzing the filter by XRF, which is much simpler than analyzing by ICPMS (Tighe, Bielski, et al., 2020).

## 5 Conclusion

A group of high‐school students from Pelham, New York, were able to access 80 local houses to test up to two samples of soil, two samples of paint, and two samples of water for Pb. No exceedances of regulatory standards for Pb were recorded in a total of 50 houses, although some samples were often missing. Among the remaining 30 houses, 45 exceedances were recorded out of the total of 305 soil, paint, and water measurements that were obtained. An established EPA model combined with the test results indicates that by far the largest risk of child exposure in the sampled houses is posed by Pb‐based paint, followed by Pb‐contaminated soil, and more rarely Pb‐contaminated water. Unfortunately, residents were also most likely to decline paint testing compared to soil and water testing. Without the involvement of students from the community, the number of paint tests might have been even lower. Pb exceedances in paint are the simplest to detect using an inexpensive commercial kit. High‐school science teachers in the many towns where child exposure remains high today should consider similar local projects for both their educational and public‐health value.

## Conflict of Interest

The authors declare no conflicts of interest relevant to this study.

## Data Availability

The de‐identified data without GPS coordinates can be downloaded from Earth and Space Open Archive at doi.org/10.1002/essoar.10507786.1.
